# Costs and cost-effectiveness of malaria reactive case detection using loop-mediated isothermal amplification compared to microscopy in the low transmission setting of Aceh Province, Indonesia

**DOI:** 10.1186/s12936-018-2361-y

**Published:** 2018-06-01

**Authors:** Brittany W. Zelman, Ranju Baral, Iska Zarlinda, Farah N. Coutrier, Kelly C. Sanders, Chris Cotter, Herdiana Herdiana, Bryan Greenhouse, Rima Shretta, Roly D. Gosling, Michelle S. Hsiang

**Affiliations:** 1 Malaria Elimination Initiative, Global Health Group, University of California, San Francisco (UCSF), San Francisco, USA; 2 Malaria Pathogenesis Unit, Eijkman Institute for Molecular Biology, Jakarta, Indonesia; 3 Department of Medicine, UCSF, San Francisco, USA; 4 Paritrana Asia Foundation, Jakarta, Indonesia; 5 United Nations Children’s Fund (UNICEF), Aceh Field Office, Banda Aceh, Indonesia; 6 Department of Pediatrics, University of Texas Southwestern Medical Center, Dallas, USA; 7 Department of Pediatrics, UCSF, San Francisco, USA

## Abstract

**Background:**

Reactive case detection (RACD) is an active case finding strategy where households and neighbours of a passively identified case (index case) are screened to identify and treat additional malaria infections with the goal of gathering surveillance information and potentially reducing further transmission. Although it is widely considered a key strategy in low burden settings, little is known about the costs and the cost-effectiveness of different diagnostic methods used for RACD. The aims of this study were to measure the cost of conducting RACD and compare the cost-effectiveness of microscopy to the more sensitive diagnostic method loop-mediated isothermal amplification (LAMP).

**Methods:**

The study was conducted in RACD surveillance sites in five sub-districts in Aceh Besar, Indonesia. The cost inputs and yield of implementing RACD with microscopy and/or LAMP were collected prospectively over a 20 months study period between May 2014 and December 2015. Costs and cost-effectiveness (USD) of the different strategies were examined. The main cost measures were cost per RACD event, per person screened, per population at risk (PAR); defined as total population in each sub-district, and per infection found. The main cost-effectiveness measure was incremental cost-effectiveness ratio (ICER), expressed as cost per malaria infection detected by LAMP *versus* microscopy. The effects of varying test positivity rate or diagnostic yield on cost per infection identified and ICER were also assessed.

**Results:**

Among 1495 household members and neighbours screened in 36 RACD events, two infections were detected by microscopy and confirmed by LAMP, and four infections were missed by microscopy but detected by LAMP. The average total cost of conducting RACD using microscopy and LAMP was $1178 per event with LAMP-specific consumables and personnel being the main cost drivers. The average cost of screening one individual during RACD was $11, with an additional cost of diagnostics at $0.62 and $16 per person for microscopy and LAMP, respectively. As a public health intervention, RACD using both diagnostics cost an average of $0.42 per PAR per year. Comparing RACD using microscopy only versus RACD using LAMP only, the cost per infection found was $8930 and $6915, respectively. To add LAMP as an additional intervention accompanying RACD would cost $9 per individual screened annually in this setting. The ICER was estimated to be $5907 per additional malaria infection detected by LAMP versus microscopy. Cost per infection identified and ICER declined with increasing test positivity rate and increasing diagnostic yield.

**Conclusions:**

This study provides the first estimates on the cost and cost-effectiveness of RACD from a low transmission setting. Costs per individual screened were high, though costs per PAR were low. Compared to microscopy, the use of LAMP in RACD was more costly but more cost-effective for the detection of infections, with diminishing returns observed when findings were extrapolated to scenarios with higher prevalence of infection using more sensitive diagnostics. As malaria programmes consider active case detection and the integration of more sensitive diagnostics, these findings may inform strategic and budgetary planning.

**Electronic supplementary material:**

The online version of this article (10.1186/s12936-018-2361-y) contains supplementary material, which is available to authorized users.

## Background

Malaria transmission in low endemic areas tends to cluster geographically and temporally. Also, in low transmission settings, a higher proportion of infected individuals is asymptomatic, and therefore will not be detected by passive surveillance which occurs through health facilities. Reactive case detection (RACD), or active case finding among households and neighbours of passively identified index cases, is a strategy to address this challenge. With the goal of RACD being the identification and treatment of asymptomatic or other infections that would not otherwise present through the passive surveillance system, it is considered a key malaria strategy for gathering surveillance information [1] and may reduce transmission and facilitate the achievement of malaria elimination [2, 3]. RACD is widely implemented [4], yet it is operationally challenging requiring significant human resources, commodities, and time for an “on-call” team to conduct screenings in villages, often traveling long distances to reach remote locations. There are also limitations with the standard diagnostics used, microscopy or rapid diagnostic test (RDT), to detect low-density infections. Highly sensitive diagnostics are available but the costs and cost-effectiveness of using them is unclear. Further, the costs of conducting RACD in general have not been systematically documented [5, 6].

The detection limit of microscopy or RDTs is typically 100 parasites/µL, and in low endemic settings, a high proportion of asymptomatic infections fall below this threshold [7, 8]. Outside of use in research settings, more sensitive detection methods such as polymerase chain reaction (PCR) are impractical due to high cost, sophisticated training, resources required, and long turnaround time (several hours). Another molecular method called loop-mediated isothermal amplification (LAMP) provides the sensitivity of PCR with fewer requirements. Testing is not point-of-care and requires use of a laboratory, but the assay is simple, does not require sophisticated equipment, and can be performed in half a day to one full day [9, 10]. However, the costs and cost effectiveness of using LAMP in RACD are not clear.

To enable national malaria programmes to assess the feasibility of implementing RACD in their low burden setting, this study aimed to: (1) estimate the cost of implementing RACD using data from the malaria-eliminating district of Aceh Besar, Indonesia; and (2) compare the cost and cost-effectiveness of using a standard diagnostic (microscopy) to more sensitive LAMP for identifying infections during RACD. This information can help guide strategic and budgetary planning for local malaria control programmes, and also help to inform the research, development, and implementation of highly sensitive diagnostics for malaria.

## Methods

### Study design

The study was a prospective economic analysis of costs and cost-effectiveness. The main cost measures were cost per RACD event, per person screened, per population at risk (PAR; defined as total population in each sub-district), and per infection found. The main cost-effectiveness measure was incremental cost-effectiveness ratio (ICER), expressed as cost per malaria infection detected by LAMP versus microscopy.

### Study location

Aceh Besar District is located in the western part of Aceh Province on Sumatra Island, Indonesia. Malaria transmission occurs year-round with the higher transmission occurring from January to July. In the past, *Plasmodium falciparum* and *Plasmodium vivax* were reported as the main malaria species, but *Plasmodium knowlesi* has also been reported from the area in 2016 [11]. Due to intensified efforts in case management, vector control, and surveillance, Aceh Besar District has successfully reduced annual malaria incidence from 2.6 cases per 1000 population in 2006 to 0.2 per 1000 in 2014 (population in 2014 was 371,412 [12]). It is one of 114 low endemic districts categorized by the Indonesian government as ‘eliminating’, with a goal to interrupt transmission by 2020 [13, 14]. As part of the malaria elimination strategy, the District Health Office in 2010 initiated RACD, which is locally referred to as ‘contact survey’. After a microscopy-confirmed malaria case is diagnosed at the health facility, designated surveillance staff visit the village of the index case to perform malaria testing among household members and neighbours. Based on WHO and national guidelines, RACD is performed using microscopy and all subjects residing within 500 m of the index case are targeted.

### Study population

Index cases were enrolled from four sub-district level health facility sites that reported 78% of all reported cases in Aceh Besar in 2013: Indrapuri, Kuta Cot Glie, Lhoong, and Saree. An additional health facility Lhoknga was also included purposively due to high case burden prior to 2013. RACD was conducted in the villages where index cases resided, and the people reached during RACD were enrolled for this study. The study was conducted over a period of 20 months from May 2014 to December 2015.

### Health facility and field procedures

All subjects presenting to health facilities with suspected malaria were assessed for malaria infection by microscopy of blood smears. Microscopy confirmed cases, also referred to as an index case, had a subsequent venous blood draw to generate a dried blood spot (DBS) for LAMP and PCR. Within one to 7 days of the index case report, RACD was conducted among individuals residing in households located within 500 m of the index cases. During an RACD event, blood was collected from individuals by finger prick to prepare slide blood smears and DBS. The RACD team consisted of five people: a microscopist and surveillance officer from the health facility, two community health workers from the village (a midwife plus a village malaria worker or village leader), and the study field coordinator. Up to two return visits were conducted to include any missing residents. The coverage goal of RACD in each of the target area was to recruit a minimum of 40 subjects or at least 80% of the residents within each of the closest five households. Additional details of index case enrolment and RACD have been previously described [11].

### Laboratory testing

The microscopists collected blood smears in the field and transported them in closed slide boxes for subsequent examination at the health facility. Slides were fixed and stained with 3% Giemsa. A slide was determined malaria positive if at least one clear form of any malaria parasite species was found after examination of the whole spread of the thick smear. Parasite densities were measured by counting the number of asexual parasites per 200 or 500 white blood cells (WBC) and calculating parasites/µL assuming a WBC count of 8000 parasites/µL in the thick smear. If positive, an additional 100 high-powered fields in the thin smear were examined to determine species [15]. Cross-check of the malaria positive slides for species identifications were done by the district level microscopist. A provincial expert level-certified microscopist did quality assurance (QA) for all positive slides, 10% of randomly selected negatives and to resolve any discrepancies in results between the clinic and district-level microscopists [13].

All DBS samples collected were dried overnight then stored in sealed plastic bags with desiccant. Extraction of DNA and LAMP testing on all samples were performed at the Aceh Provincial Health Laboratory. DNA was extracted from DBS using the Saponin/Chelex-100 method [16]. Using 15 µL of chelex-extracted DNA solution, Pan-LAMP testing followed by Pf-LAMP specific testing for Pan-LAMP positive samples was performed using a commercial Loopamp detection kit [17, 18] in accordance to manufacturer’s instructions (Eiken Chemical, Japan) (Limit of detection (LOD) between 1 and 5 parasites/µL). As previously detailed, using DNA chelex-extracted from a second DBS, Pan-LAMP positive and 10% of Pan-LAMP negative samples underwent further molecular testing for QA at the Eijkman Institute for Molecular Biology using nested PCR methods (LOD between 0.1 and 1 parasites/µL) targeting the *cytochrome b* gene followed by *Alu*I enzyme digestion for species identification and 18S rDNA nested PCR, with positive samples undergoing further *P. knowlesi*-specific PCR testing [11].

### Costing data collection

Costs were collected for RACD programme set-up, training, coordination, and all activities beginning from when an index case was reported from a health facility (e.g. preparing supplies and contacting the index case household) through to sample processing, analysis, and quality assurance conducted after the RACD event. Time spent by personnel on microscopy or LAMP, including the quality assurance by subsequent microscopy and PCR, respectively, was allocated to diagnostic-specific costs as appropriate. Follow-up costs including a return visit to provide treatment to subjects that were initially microscopy-negative but subsequently found to be LAMP-positive were not included.

Data on costs were collected by a local field study coordinator through review of expenditure records. All costs were converted from the local currency to US dollars (USD) using the average mid-year exchange rate for 2015 (1 USD = 13,432 Indonesian Rupiah, from Oanda.com). These data were inputted to a costing tool developed in Microsoft Excel 2010. Costs were organized by location at which the expenses occur (health facility, provincial laboratory, and national laboratory); the type of diagnostic test used (microscopy or LAMP); and the month in which the cost was incurred. Each cost input was grouped into one of five major categories.

#### Personnel

Personnel costs covered project staff salaries for trainings, coordination, field activities and laboratory work. Project staff included one full time field coordinator, a part time field team in each of the five sub-districts including one surveillance officer and a microscopist, three part-time laboratory technicians at the provincial and national levels, and one laboratory technician at the national level. Most of the personnel had duties outside the scope of this study, and only supported RACD as index cases presented to health facilities. Thus, specific time contributions of all personnel involved in the RACD activities were logged and later used for approximating the cost of human resources. Time required for initial diagnosis of index cases at health facilities was not included.

#### Trainings

Costs associated with any trainings conducted for this study were captured and include costs for training supplies, room rental, and participant per diems.

#### Services

The cost of services included utilities such as internet, communication, courier services, and vehicle rental/fuel for sample transportation used in conducting RACD. Services utilized prior to the study period but for project setup were included.

#### Capital

Capital costs were initial investments to set up RACD and included the cost of owned motor vehicles used for travel and transportation; electronic devices such as computers, computer accessories, printers, tablets, refrigerators; and laboratory equipment such as microscopes, centrifuges, heat blocks, and PCR machines. If the capital was used for activities other than RACD, a time use percentage was assigned. The cost of capital was valued for the study duration (20 months) after accounting for depreciation based on the useful life years of each capital, and discounted using a rate of 3%. The remaining values of capital at the end of the study period were subtracted. The cost of office space and furniture were not included due to limitations in data availability.

#### Consumables

Consumables included field and laboratory supplies such as slides, lancets, alcohol swabs, plastic bags, filter paper, LAMP kits, laboratory reagents, centrifuge tubes, etc. Per unit costs of these consumables were multiplied by the amounts utilized during the study period to estimate the total cost of consumables.

### Analysis

The main outcome measure was number of infections detected by LAMP versus microscopy in RACD. Other measures included number of RACD events and number of individuals screened per RACD event.

To estimate the costs associated with microscopy versus LAMP, RACD costs were separated into ‘general RACD costs’ and ‘diagnostic-specific costs’. General RACD costs covered routine activities related to RACD such as the field visit, including time for preparation, travel, and screening households. Diagnostic specific costs for microscopy and LAMP were calculated by isolating any personnel, training, consumables, processing time, services from general RACD costs. As a key activity for an elimination surveillance programme, particularly for programmes considering the use of highly sensitive diagnostics, QA was included for both microscopy- and LAMP-specific costs [1]. Trainings for microscopy and microscopy QA were conducted in conjunction with the surveillance trainings and included in the general RACD training costs. Cost proportions for each input category were compared for microscopy-specific, LAMP-specific and general RACD costs.

Cost per RACD event and cost per individual screened were calculated by dividing the total cost of conducting RACD using microscopy and/or LAMP during the study period by the total number of RACD events and screened individuals. Cost per population at risk (PAR; defined as total population in each sub-district) was calculated at the sub-district level. Cost per PAR per year was calculated by dividing the total cost of RACD using both diagnostics by the population and by the number of months the study was conducted (20 months). The resulting montly cost per PAR, was then multiplied by 12 (months in a year) to determine the cost per PAR per year. Costs per infection identified were calculated as cost of RACD using microscopy and/or LAMP, divided by the number of infections detected by microscopy and/or LAMP, respectively. The total cost for the study period and annual recurrent programmatic cost of the RACD programme were also calculated. The annual recurrent programmatic cost was approximated by excluding any capital non-recurrent costs from the total cost.

To compare the cost-effectiveness of the different diagnostic methods, the incremental cost-effectiveness ratio (ICER), defined by the difference in cost between two interventions divided by the difference in their effect, was estimated by comparing the cost and outcomes associated with LAMP and microscopy. As general RACD costs were applied to both methods, the differences in costs were due to diagnostic-specific costs and respective yield.

To explore how costs might vary across sites with different index case burdens or prevalence of infection in RACD [19], cost measures were compared across the sub-districts. The cost drivers of RACD across the study sites were also assessed. To consider how changes or differences in diagnostic sensitivity or prevalence of infection in RACD might affect findings, the costs and ICER for different scenarios was estimated based on the costs and outcomes observed in this study.

## Results

### Enrollment and outcome measures

**Fig. 1:**
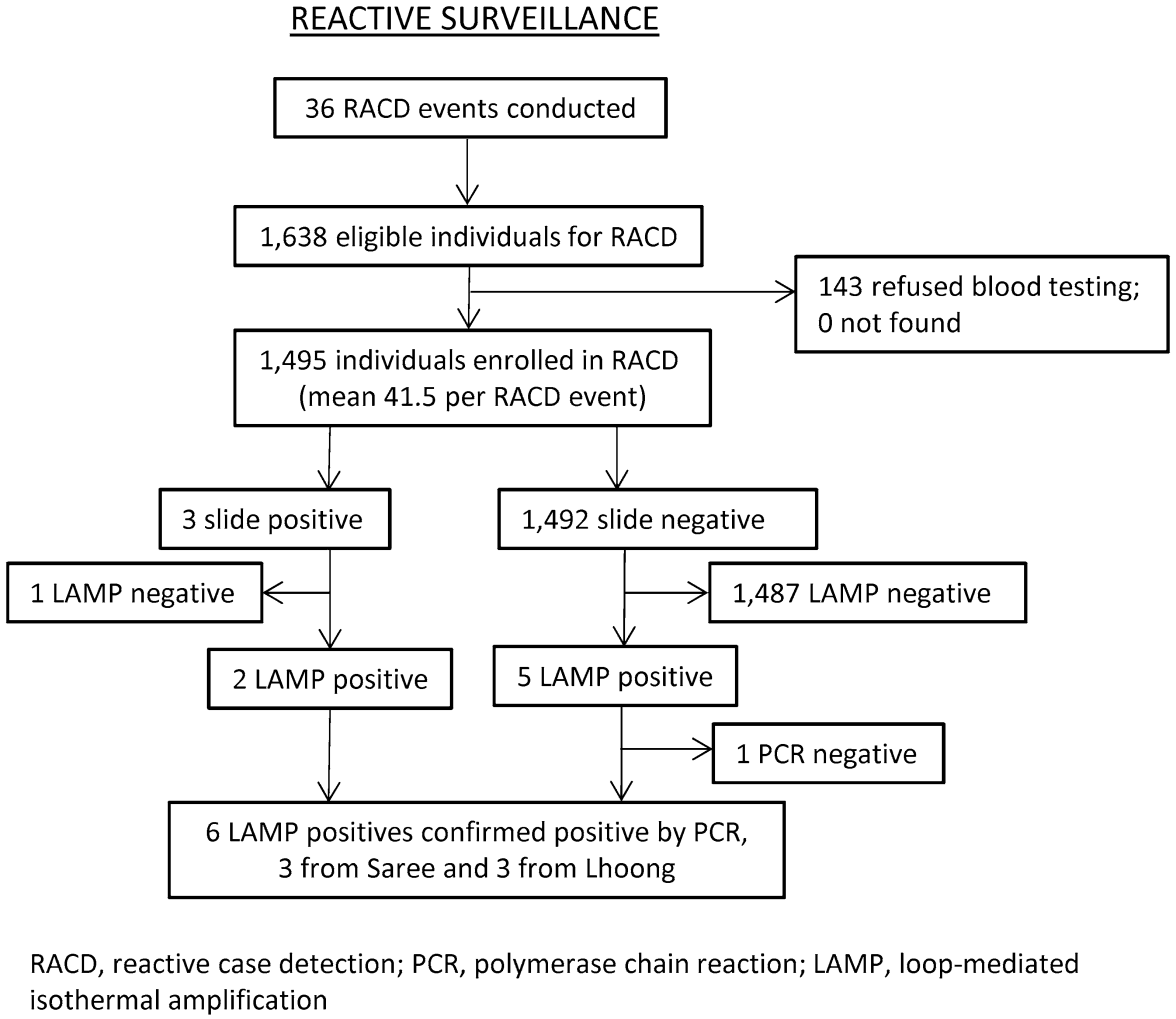
Enrollment in reactive case detection (RACD) with laboratory results

Study enrollment and laboratory reports have previously been reported [11], but are shown in Fig. 1 for reference. In brief, a total of 36 eligible positive index cases were identified in the health facilities triggering 36 RACD events. Of the 1638 eligible individuals residing in the screening radius of the index cases, 1495 (91%) were enrolled in the study. On average, about 42 individuals were screened or tested per RACD event. Of these individuals tested during RACD, a total of eight additional infections were identified. Three of these additional cases were initially found to be microscopy positive, but one was determined to be a false positive as confirmed by LAMP. An additional five individuals, originally found to be microscopy negative, were positive when tested by LAMP. Thus, seven additional infections found via RACD were confirmed LAMP positives. When the seven LAMP positives underwent PCR for quality assurance, one was negative. In total, six PCR-confirmed cases were identified, with three each from Saree and Lhoong study sites, and were classified as three *P. vivax*, two *P. falciparum*, and one *P. knowlesi*. The remaining three sites (Kuta Cot Glie, Indrapuri, and Lhoknga) identified no additional infections as a result of RACD. A summary of the outcome measures (number of RACD events, subjects screened, and infections identified) for the overall study and by sub-district is shown in Table 1.

**Table 1:** Summary of outcome measures, total costs, and cost effectiveness of RACD using microscopy and LAMP by sub-district over study period

Sub-district	Population at risk	Outcome measures			Total cost	Average costs			
		No. of RACD events	No. of subjects screened	No. infections identified (%)	Total	Per PAR per year	Per RACD event	Per individual screened	Per infection identified
Lhoong	9592	14	511	3 (0.6%)	$12,703	$0.79	$907	$25	$4234
Saree	11,346	12	527	3 (0.6%)	$12,748	$0.67	$1062	$24	$4249
Kuta Cot Glie	10,560	8	377	0 (0%)	$9657	$0.55	$1207	$26	–
Indrapuri	14,052	2	80	0 (0%)	$4391	$0.19	$2196	$55	–
Lhoknga	15,659	0	0	0 (0%)	$2918	$0.11	–	–	–
Total	61,209	36	1495	6 (0.4%)	$42,418	$0.42^a^	$1178	$28	$7070

Population at risk is defined as total population of sub-district

*LAMP* loop-mediated isothermal amplification, *RACD* reactive case detection

^a^Sub-district average cost per PAR

### Overall cost of RACD using microscopy and LAMP

The total cost of conducting RACD using microscopy and LAMP during the study period was $42,418 (Table 1). On average, the cost per RACD event was estimated to be $1178 and the cost per individual screened was $28. The average cost per infection identified was $7070. As a public health intervention, the annual cost per PAR was estimated at $0.42 averaged across the sub-districts.

### Cost and cost-effectiveness by microscopy versus LAMP

**Fig. 2:**
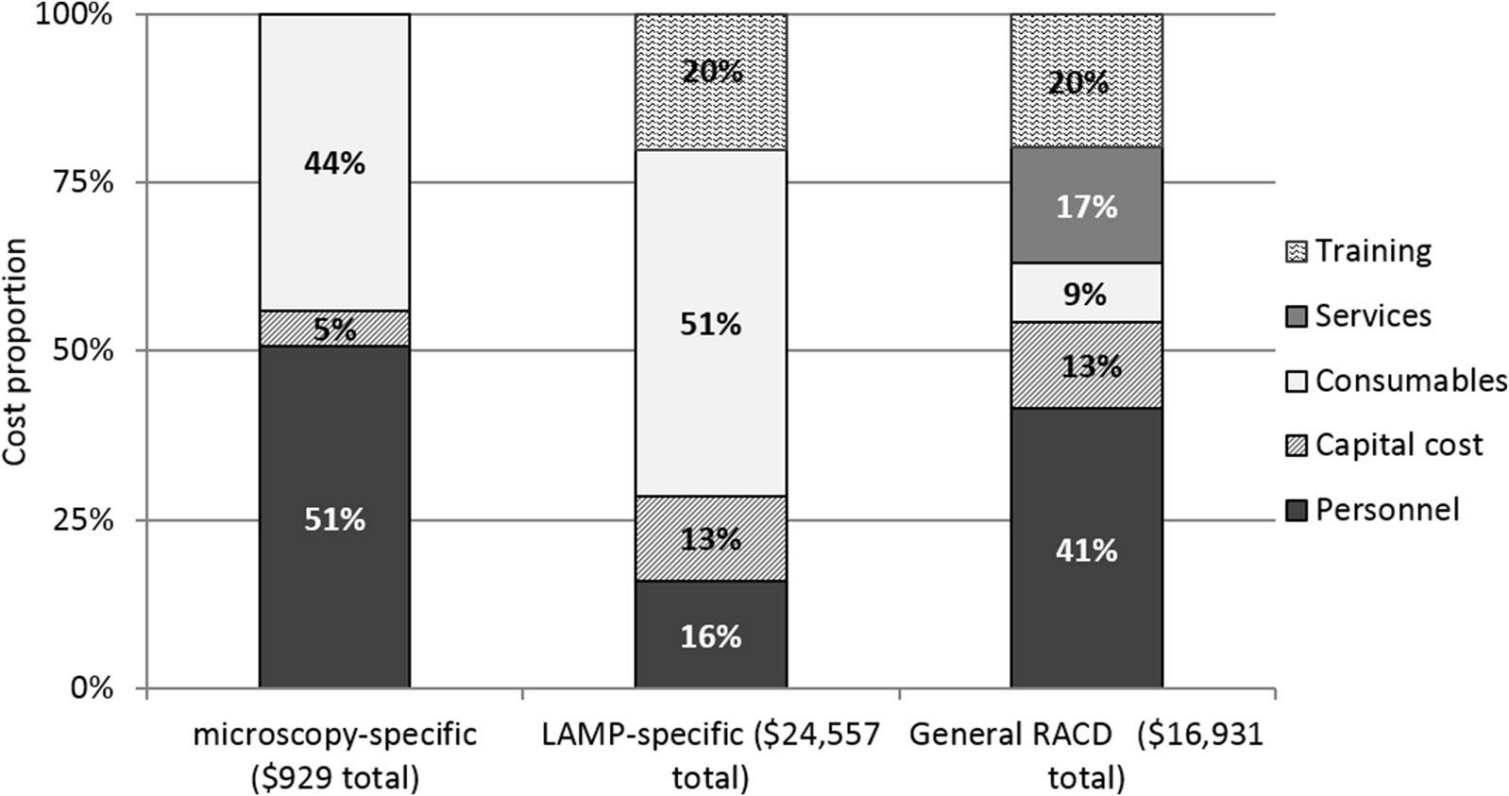
Cost proportions by input category for general RACD, microscopy-specific, and LAMP-specific activities over study period

Microscopy-specific costs, which consists of consumables, such as slides and Giemsa, personnel time used to process and read the slides, microscopes, and staff training, was calculated at $929 in total, which translated to $0.62 per individual screened (Table 2). LAMP-specific costs, which includes LAMP-specific capital and consumables (kits, DBS, reagents, and laboratory equipment for QA) as well as trainings and personnel time to process and run the blood samples for LAMP with quality assurance, totaled $24,557 over the course of the study. This is equivalent to $16 per individual screened. Consumables, followed by training and personnel costs were the main cost drivers for LAMP (Fig. 2). Compared to a box of 100 microscopy slides, which were purchased for $4.50 (equivalent to $0.05 per test), Pan-LAMP tests cost $3.75 each and Pf-LAMP tests cost $11.97 each. Reagents used for PCR for LAMP QA also constitute to a major share of consumables. General RACD costs, which could not be attributed to either diagnostic specifically, totaled $16,931, or $11 per individual screened. Table 2 also presents costs for overall RACD costs with microscopy and LAMP, RACD with microscopy only, and RACD with LAMP only over the course of the study period, as well as annualized recurrent costs for RACD, microscopy, and LAMP specific costs. The annual recurrent costs, or costs which exclude one-time purchase capital costs, are indicative of the costs that the programme can expect for each year that the intervention is implemented. For example, a programme that has all the necessary capital equipment for LAMP, such as a heat block and PCR machine, can expect to budget $9 per person screened by LAMP, in addition to the annual recurrent cost of RACD.

**Table 2:** Summary of costs by outcome measure

	General RACD costs	Microscopy-specific costs	LAMP-specific costs	Overall RACD (microscopy and LAMP)	Overall RACD (microscopy only)	Overall RACD (LAMP only)	Annual recurrent general RACD costs	Annual recurrent microscopy-specific costs	Annual recurrent LAMP-specific costs
Total costs (36 RACD events)	$16,931	$929	$24,557	$42,418	$17,860	$41,489	$6957	$527	$12,871
Average cost per RACD event	$470	$26	$682	$1178	$496	$1152	$193	$14.65	$358
Average cost per individual screened	$11	$0.62	$16	$28	$12	$28	$5	$0.35	$9
Average cost per infection identified	$2822	$465	$4093	$7070	$8930	$6915	$1159	$264	$2145

*RACD* reactive case detection, *LAMP* loop-mediated isothermal amplification

Cost shares by input category were compared between the diagnostic methods (LAMP and microscopy), as well as to general RACD costs (Fig. 2). Personnel costs accounted for the major share for general RACD and microscopy specific costs, at 41 and 51%, respectively. Consumables accounted for the largest share of LAMP specific costs at 51%, and second largest share for microscopy at 44%. Training cost was the second largest as a share of cost for LAMP and general RACD at 20% each. Four trainings were held between September 2013 and January 2015. Of the four, one training was LAMP-specific laboratory training. The remaining three focused on general RACD protocols, and included refreshers on blood collection techniques for microscopy and LAMP. Personnel time and training were also the main cost drivers for RACD across each study site.

The share of capital costs for LAMP-specific and general RACD activities was at 13% each. A more detailed breakdown of all capital costs can be found in the annex (Additional file 1: Figure S1). Of all capital costs, 61% was from laboratory equipment mainly for LAMP and LAMP QA followed by tablets and accessories (33%). Eight of the ten highest capital costs, ranging from $82 to $1542, were attributed to LAMP or LAMP QA and included laboratory equipment such as a heat block, Gel doc system, a thermomixer, a UV sterilization cabinet, a refrigerator, a centrifuge, and a PCR machine, though all of these items except the heat block and pipette were only used for RACD between 15 and 75% of the time. Usage of the gel doc system, thermomixer, UV Sterilization cabinet, and PCR machines are not crucial for LAMP detection itself, but are important for LAMP QA and represent a large share of the capital cost. Two of the top ten high value capital were attributed to general RACD costs and included seven tablets used for data collection and study laptops. The remaining capital items used for RACD had attributed costs ranging from $1.75 to $74. Additional file 1: Tables S1, S2 provide a more detailed breakdown of input costs by location and cost category.

Two infections were identified among all individuals tested with microscopy. The resulting cost per additional infection identified by RACD using microscopy was estimated to be $8930, where the microscopy-specific cost accounted for only about 5% (or $465 per infection). Six infections were identified via LAMP through RACD at a cost of $6915 each (LAMP-specific cost of $4093 per infection). The ICER of detecting an infection through LAMP compared to microscopy was estimated to be $5907 per infection.

### Costs by sub-district

Of the five cost categories, LAMP-specific consumables were the largest cost driver at 34% of the total expense for all sub-districts. Personnel and trainings accounted for the next largest shares of the expense at an average of 27 and 20%, respectively. Capital and services accounted for an average of 13 and 7%, respectively, across the sub-districts. As Lhoknga had no index cases, no services or consumables costs were incurred, which resulted in a larger proportional share of training and capital costs (57 and 37%, respectively).

The distribution of total costs for RACD using microscopy and LAMP varied across the study sites (Table 1). In Lhoong and Saree, which had the highest number of RACD events and individuals screened, the total cost was the highest of the five study sites at $12,703 and $12,748 respectively, and the cost per individual screened was between $25 and $24 per person screened each. With eight RACD events in Kuta Cot Glie, total cost was lower than Saree and Lhoong, with cost per individual screened being similar ($26). With two RACD events, Indrapuri had the highest cost per individual screened at $55. Lhoknga had no RACD events and thus no cost per individual screened, but the site maintained a cost of $4391, primarily due to personnel (including training) and a small amount of capital requirements to be prepared to launch RACD if needed. The cost per PAR per year by sub-district ranged from $0.11 in Lhoknga to $.79 in Lhoong, and averaged $0.42 across the total population of the five sub-districts (Table 1).

### Effect of infection prevalence on costs and cost-effectiveness

**Fig. 3:**
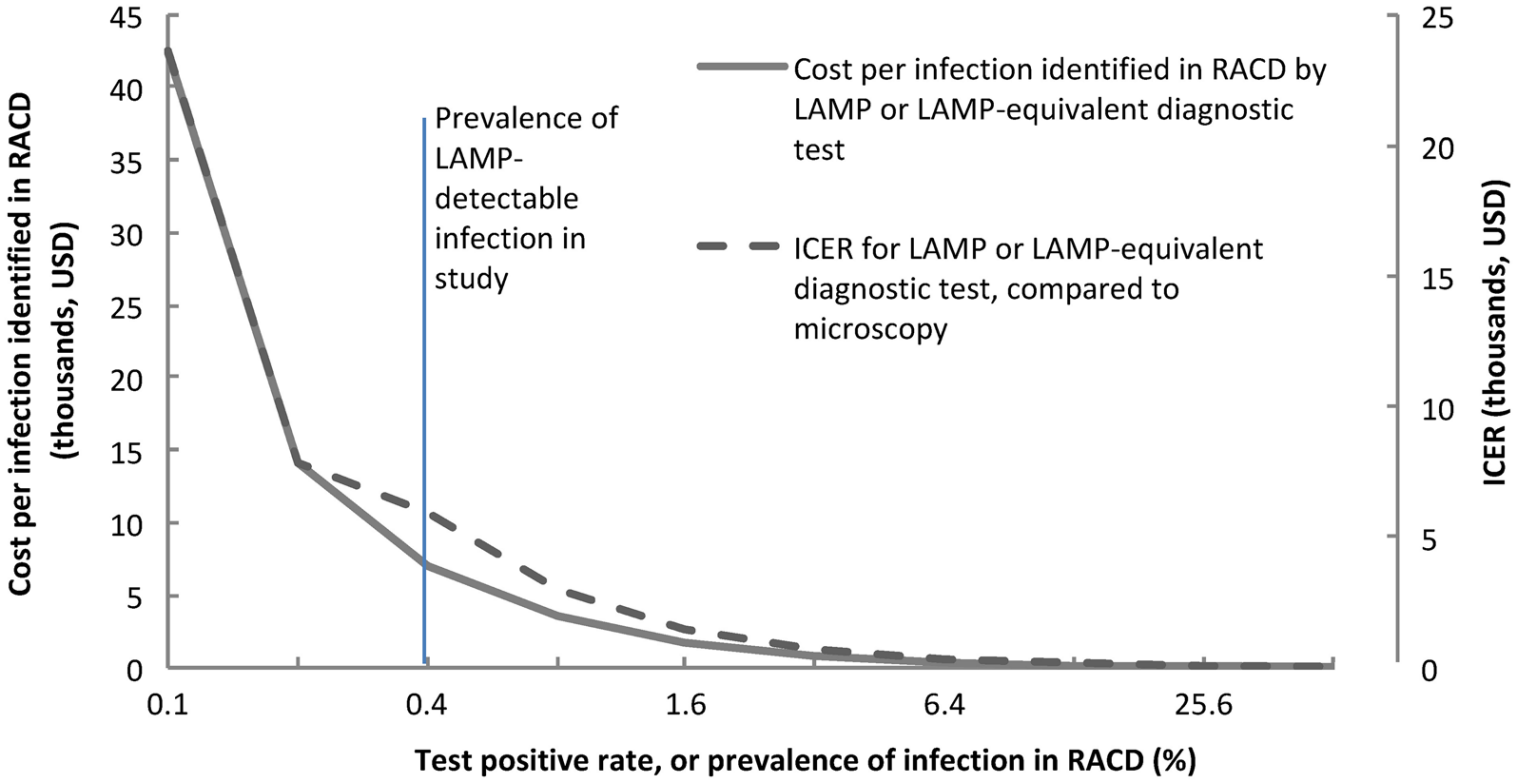
Estimated costs per infection identified in RACD and incremental cost-effectiveness ratio (ICER) of infections identified in using LAMP versus microscopy, by prevalence of LAMP-detectable infection in RACD, assuming same general and per unit costs and 33.3% sensitivity of microscopy compared to LAMP as observed in study

For settings with lower or higher prevalence of infection compared to Aceh Besar, the costs using LAMP or another equivalent test with 3:1 diagnostic yield relative to microscopy were estimated (Fig. 3, solid line). At 0.4% prevalence of infection, the cost per infection identified is $7070, and declines to $1767 when the prevalence is 1.6%. Cost declines begin to plateau thereafter. The impact of prevalence of infection on ICER was also estimated (Fig. 3, dotted line). As the calculation for ICER is based on cost per infection, ICER also declines with increasing prevalence of infection. At 0.4% prevalence of infection, ICER was $5907, and declines to $1477 when the prevalence of infection is 1.6%. The ICER decline slows and begins to plateau thereafter.

### Effect of diagnostic yield on costs and cost-effectiveness

**Fig. 4:**
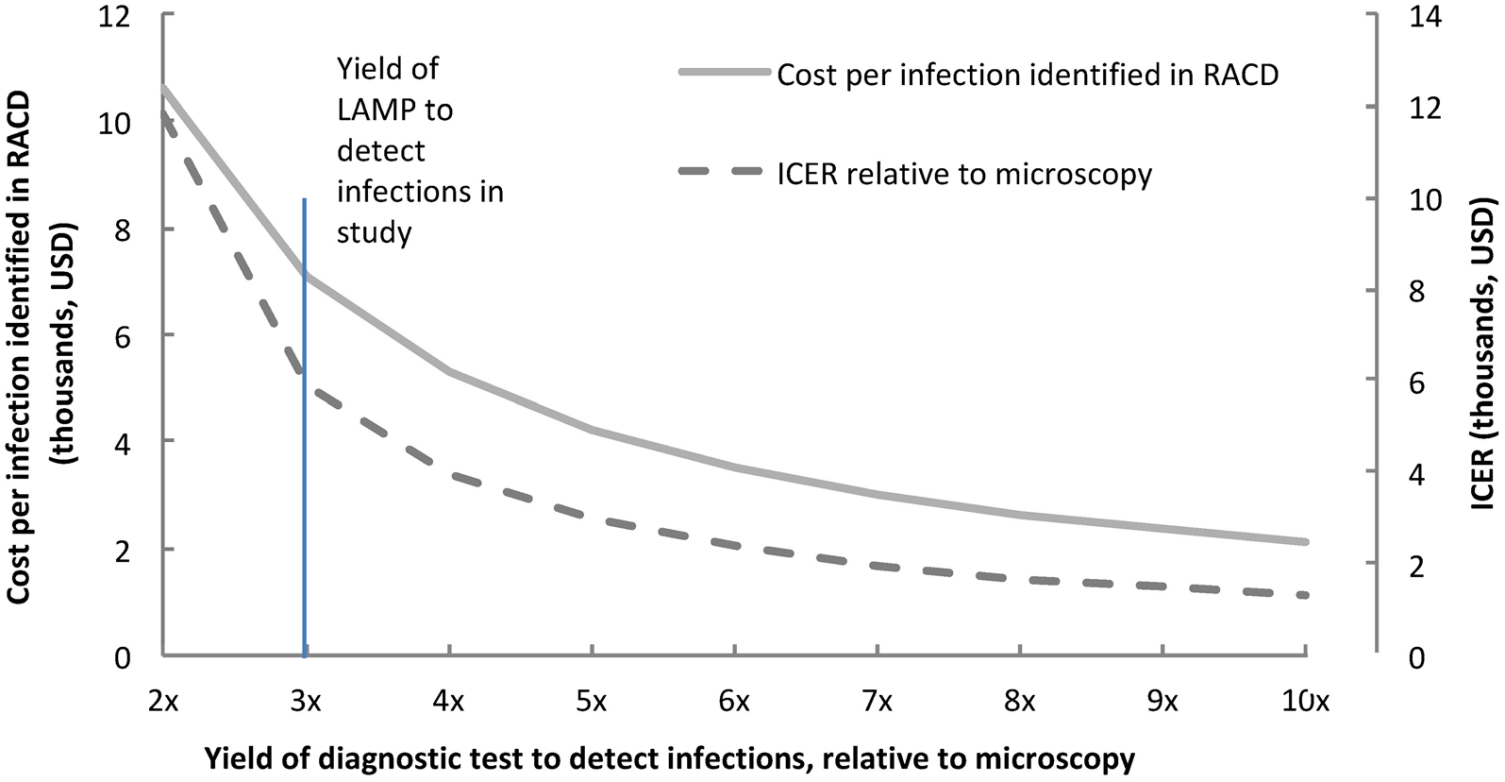
Estimated costs per infection identified in RACD and incremental cost-effectiveness ratio (ICER) by yield of diagnostic test to detect infections relative to microscopy, assuming same general and per unit costs as LAMP and same 0.13% prevalence of microscopy-detectable infections as observed in study

Although LAMP detected threefold more infections than microscopy, our sample size was small. Other diagnostics, existing or in development, may provide a different yield relative to microscopy [20]. Assuming prevalence of microscopy-detectable infection to be 0.13% and base costs similar to LAMP, the impact of diagnostic yield on costs per infection identified and ICER was estimated (Fig. 4). When the yield is three fold relative to microscopy, the cost per infection identified is $7070 (as in our study). When the yield increases to fivefold, the costs decreases to $4242, with the curve nearing a plateau when the yield reaches tenfold, where costs are $2121 per infection identified. The ICER follows the same trend. At a yield of three fold relative to microscopy, the ICER is $5907 and it continues to reduce to $2954 when the yield is fivefold and $1313 when the yield is tenfold relative to microscopy. The same trends would apply to a situation with a higher proportion of low-density infection (e.g. as malaria transmission declines).

## Discussion

RACD is a strategy widely implemented to gather surveillance information in low transmission settings and it may contribute to reductions or interruption of transmission. However, the costs of doing RACD have not been well evaluated. This study investigated the cost of conducting RACD and the cost-effectiveness of different diagnostic methods in identifying additional malaria infections. During the study period of 20 months, 1495 individuals were screened in 36 RACD events yielding six additional cases by microscopy or LAMP, at an estimated total cost of $42,418. The average cost of conducting RACD using microscopy and LAMP was $1178 per event, with the main cost driver being personnel. The average cost of RACD for each individual was $11 for general costs, with an additional $0.62 and $16 per person for microscopy and LAMP, respectively. As a public health intervention, RACD using both diagnostics cost an average of $0.42 per PAR per year. LAMP was more costly but more cost-effective for the detection of infections mainly due to higher diagnostic sensitivity. For RACD using microscopy only, the cost per infection found was $8930 compared to $6915 for RACD using LAMP only. The ICER for RACD using LAMP versus microscopy was $5907 per additional malaria infection detected. As malaria programmes consider active case detection and the integration of highly sensitive diagnostics, these findings may inform strategic and budgetary planning.

Due to the intensified interventions required, it is well-established that malaria elimination compared to control is more costly but that longer term benefits make the pursuit worthwhile [21, 22]. A systematic review on the costs and cost-effectiveness of control interventions by White et al. [23] found the median cost of preventative interventions (insecticide-treated bed nets, indoor residual spraying, intermittent preventative treatment) was $0.60–$6.70 per person and the median costs for case management services (diagnosis, treatment) ranged from $4.32–$30.26 per patient, with the most expensive service being treatment of severe malaria. Our finding that RACD with microscopy and/or LAMP cost $12–$28 per individual screened is consistent with previous reports on the higher cost for elimination versus control interventions. The high cost of $7070 per infection identified also raises the issue of the value of RACD as a low yield activity where case detection rates are generally less than 2% even with highly sensitive diagnostics [24]. However, the value of an intervention should be considered in a broader context. As a public health intervention, the annual cost of RACD using both diagnostics was low, at an average of $0.42 per PAR at the sub-district level.

Despite the high costs, RACD using LAMP versus microscopy led to a lower cost per infection identified (23%) and the ICER of $5907 per additional infection detected favors the use of LAMP or a LAMP-equivalent assay in RACD. Based on our analyses of cost inputs, there would opportunities to mitigate costs. Personnel and LAMP-specific consumables, and not capital, training, or services, accounted for the highest cost shares [25]. Personnel costs could be minimized by better integrating staff in the health system. LAMP-specific consumable costs could be decreased if prices were negotiated or  subsidized, or a less expensive highly sensitive diagnostic could be used. In addition, the higher sensitivity of LAMP made it possible to detect *P. knowlesi* in the study area for the first time. This important benefit may outweigh the high costs of RACD using LAMP.

To explore how costs might vary across sites with different index case burdens or prevalence of infection in RACD, cost measures were compared across the sub-districts. In sub-districts with more RACD events (such as Saree and Lhoong) overall costs were higher than in sub-districts with fewer cases, and consequently the cost per individual screened was lower. Despite having zero cases during the study period, Lhoknga still needed to maintain costs for training “on-call” local health facility personnel as well as capital in the event an index case were to present and RACD would need to be conducted.

To further inform how costs and cost-effectiveness might vary with differences in prevalence of infection or the sensitivity of the diagnostic used, estimated projections show how costs per infection identified along with ICER decrease with increases in prevalence of infection and yield of a diagnostic to detect infections, relative to microscopy (Figs. 3 and 4). As seen in the Fig. 3, the curve began to plateau when the test positive rate or prevalence of infection in RACD increased above 1.6% suggesting that the highest relative cost-savings will be realized in low transmission settings. The curve also began to plateau when the yield of the diagnostic test exceed fivefold–tenfold relative to microscopy, suggesting diminishing returns with use of more sensitive diagnostics [26] when the prevalence is already low. As more sensitive diagnostics are being developed or come to market, these considerations can help to inform decision-making on investments and strategic planning.

There were some limitations of this study. Estimation of the personnel costs relied on self-reported time allocations. Further, due to the few cases in this low transmission setting, limitations exist for the generalizability of the study and precision of the ICER estimate. Extrapolations to consider the influence of prevalence of infection and diagnostic yield were performed, but assumed other fixed factors (epidemiological, or cost-related). In real world implementation, several relevant factors could change and affect costs. For example, cost-effectiveness of LAMP could be improved with discounted prices for LAMP or other less costly diagnostics (e.g. a new highly sensitive histidine-rich protein 2-based rapid diagnostic test [27] could be useful in *P. falciparum* predominant settings and has lower costs due to a discounted price and does not require laboratory work nor travel back to the households to inform on results). In a more remote and underserved setting, there may be higher costs associated with establishing laboratory infrastructure to conduct LAMP. A new diagnostic may have higher costs than those required for LAMP. These scenarios would increase costs per infection identified and reduce cost-effectiveness. Also, the estimations assume very low parasite densities and do not take into account that in other settings, due to endemicity or species (e.g. *P. falciparum* presents with higher parasite densities than *P. vivax*), parasite densities may be different and thus impact the sensitivities and yield of microscopy and LAMP. More empiric data from other settings along with use of more sophisticated modeling techniques could improve generalizability. Additionally, while microscopy and LAMP both require time to acquire results, processing time for LAMP may be longer resulting in a delay in treatment and therefore transmission-blocking, which were not taken into consideration in this analysis. Finally, this study only assessed costs from service provider’s perspective, including cost sharing and in-kind donations, and costs are specific to this situation. In other contexts, absolute costs may be different. Future studies can build upon these findings to quantify costs and benefits of conducting RACD by including the broader costs to society when additional infections are not detected and may result in further transmission.

This study had several strengths. Firstly, a detailed and prospective collection of RACD costs was carried out. Retrospective data collection can introduce bias and limit the granularity of the costing data. Importantly, this study fills a critical gap on the economics of focal screen and treat. Previous studies have documented a framework for evaluating the costs of RACD [25], conducted cost analyses of mass screen and treat [28], or identified potentials for operational efficiencies [29], but only one other study has measured the actual costs and that study was from a higher transmission setting where the RACD test positivity rate was 19% [25]. To the best of the authors’ knowledge, this study is the first on RACD or focal screening and treatment to report costs from a low transmission setting, costs using a highly sensitive diagnostic, and cost-effectiveness of using a highly sensitive versus standard diagnostic in active case detection.

In summary, in the low transmission setting of Aceh Besar, RACD costs per individual screened were found to be high, though costs per PAR were low. Compared to microscopy, the use of LAMP in RACD was more costly but more cost-effective for the detection of infections, with diminishing returns observed when findings were extrapolated to scenarios with higher prevalence, or using more sensitive diagnostics when prevalence is very low. These findings can inform strategic and budgetary decisions faced by the many countries that are pursuing malaria elimination and considering active case detection with new diagnostics [1, 30, 31].

## Additional file

**Additional file 1: Figure S1.** Cost proportion breakdown of capital costs. **Table S1.** Detailed summary of input costs (USD) by location. **Table S2.** Detailed summary of input costs (USD) by cost category. **Table S3.** Detailed list of top ten capital costs, discounted annually at 3% from year of purchase (USD).
