# Supplementary material for: Costs and cost-effectiveness of malaria reactive case detection using loop-mediated isothermal amplification compared to microscopy in the low transmission setting of Aceh Province, Indonesia
Source: Malar J. 2018 Jun 1;17:220. doi: 10.1186/s12936-018-2361-y (PMC5984760; doi:10.1186/s12936-018-2361-y)
Supplement: Supplementary file 1 — Additional file 1: Figure S1. Cost proportion breakdown of capital costs. Table S1. Detailed summary of input costs (USD) by location. Table S2. Detailed summary of input costs (USD) by cost category. Table S3. Detailed list of top ten capital costs, discounted annually at 3% from year of purchase (USD). [file 12936_2018_2361_MOESM1_ESM.docx]

Additional file 1

Figure S1. Cost proportion breakdown of capital costs


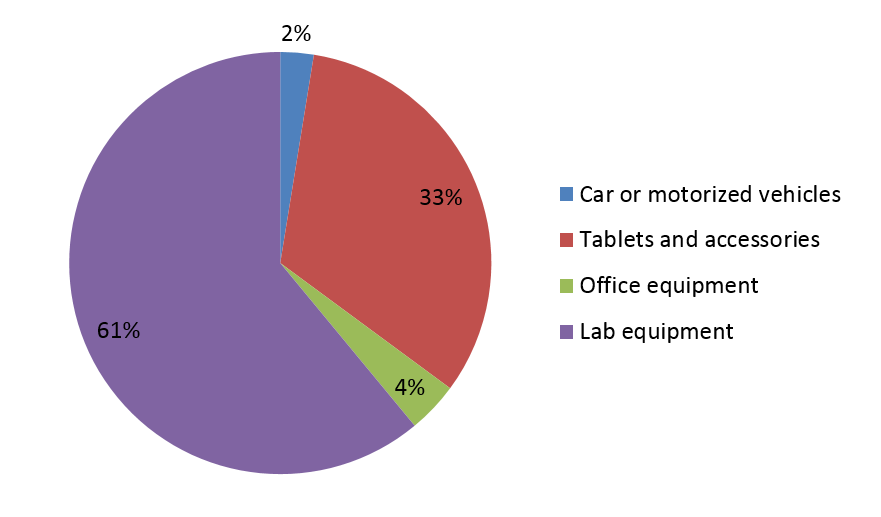


Table S1. Detailed summary of input costs (USD) by location

| **Capital** | **General RACD** | **Microscopy-specific** | **LAMP-specific** | **Total** |
| --- | --- | --- | --- | --- |
| **Provincial lab cost** | 383 | 50 | 936 | 1,369 |
| **Central Lab cost** | 0 | 0 | 2,052 | 2,052 |
| **Health facility cost** | 1,797 | 0 | 118 | 1,915 |
| **Total** | **2,180** | **50** | **3,106** | **5,336** |
|  |  |  |  |  |
| **Consumables** | **General RACD** | **Microscopy-specific** | **LAMP-specific** | **Total** |
| **Provincial lab cost** | 83 | 81 | 6,976 | 7,140 |
| **Central Lab cost** | 149 | 0 | 4,932 | 5,081 |
| **Health facility cost** | 1,240 | 327 | 657 | 2,224 |
| **Total** | **1,471** | **409** | **12,566** | **14,445** |
|  |  |  |  |  |
| **Services** | **General RACD** | **Microscopy-specific** | **LAMP-specific** | **Total** |
| **Provincial lab cost** | 2,239 | 0 | 0 | 2,239 |
| **Central Lab cost** | 179 | 0 | 25 | 204 |
| **Health facility cost** | 488 | 0 | 0 | 488 |
| **Total** | **2,905** | **0** | **25** | **2,930** |

Table S2. Detailed summary of input costs (USD) by cost category

|  | **General RACD** | **Microscopy-specific** | | **LAMP-specific** | | **Total** | |  |  |
| --- | --- | --- | --- | --- | --- | --- | --- | --- | --- |
| **Capital*** | | | | | | | |  |  |
| Car or motorized vehicles | 129 | 0 | | 0 | | 129 | |  |  |
| Tablet and accessories | 1,650 | 0 | | 0 | | 1,650 | |  |  |
| Office equipment | 401 | 0 | | 65 | | 466 | |  |  |
| Lab equipment | 0 | 50 | | 3,041 | | 3,091 | |  |  |
| **Total** | **2,180** | **50** | | **3,106** | | **5,336** | |  |  |
| ***** See Table A3 for more detailed information on top ten capital costs, including indication of whether equipment was used quality assurance (QA) | | | | | | | |  |  |
|  |  |  | |  | |  | |  |  |
|  | **General RACD** | **Microscopy-specific** | | **LAMP-specific** | | **Total** | |  |  |
| **Consumables**  **Total** | **1,471** | **409*** | | **12,566**** | | **14,445** | |  |  |
| *****90% of the cost is for conducting microscopy and 10% is for microscopy QA.  ****** 90% of the cost is for conducting LAMP and 10% is for LAMP QA (PCR). | | | | | | | |  |  |
|  |  |  |  |  |  |  |  |  |  |
| **Services** | **General RACD** | | **Microscopy-specific** | | **LAMP-specific** | | **Total** |  |  |
| Electronics/Information Technology | 712 | | 0 | | 0 | | 712 |  |  |
| Transport^ | 2,193 | | 0 | | 25 | | 2,218 |  |  |
| **Total** | **2,905** | | **0** | | **25** | | **2,930** |  |  |
|  |  | |  | |  | |  |  |  |
|  | **General RACD** | | **Microscopy-specific** | | **LAMP-specific** | | **Total** |  |  |
| **Trainings**  **Total** | **3,362** | | **0*** | | **4,949** | | **8,311** |  |  |
| ^ Samples for microscopy and LAMP were transferred simultaneously to the lab by the RACD teams and therefore included in the general RACD costs.  * Trainings for microscopy and microscopy QA were conducted in conjunction with the surveillance trainings and included in the general RACD training costs. | | | | | | | |  |  |
|  |  | |  | |  | |  |  |  |
| **Personnel staff salary** | **General RACD** | | **Microscopy-specific** | | **LAMP-specific** | | **Total** | **# staff** | **Average % per month attributed to RACD** |
| Coordinator | 2,090 | | 2,090 | | 2,090 | | 6,269 | 1 | 63% |
| Surveillance officer | 248 | | 248 | | 248 | | 743 | 5 | 3% |
| Microscopist | 0 | | 470 | | 0 | | 470 | 5 | 5% |
| Lab tech 1 (Aceh) | 0 | | 0 | | 882 | | 882 | 5 | 4% |
| Lab tech 2 (Eijkman) | 0 | | 0 | | 3,030 | | 3,030 | 1 | 51%* |
| **Total** | **2,337** | | **2,808** | | **6,249** | | **11,395** |  |  |

*Of the 51% of the Lab tech (Eijkman) time that is attributed to RACD, 20% is for conducting LAMP and 31% is for LAMP QA (PCR).

Table S3. Detailed list of top ten capital costs, discounted annually at 3% from year of purchase (USD)

| **Item** | **Quantity** | **% of time attributed to RACD**** | **Total cost attributed to RACD** | **Activity** |
| --- | --- | --- | --- | --- |
| Tablets | 7 | 100% | 1,542 | General RACD |
| Gel doc System***** | 1 | 15% | 785 | LAMP-specific |
| Heat block | 3 | 100% | 656 | LAMP-specific |
| Pipette | 2 | 100% | 483 | LAMP-specific |
| Thermomixer***** | 1 | 15% | 355 | LAMP-specific |
| Laptop | 1 | 100% | 250 | General RACD |
| UV sterilization Cabinet***** | 1 | 15% | 212 | LAMP-specific |
| Refrigerators***** | 5 | 75% | 118 | LAMP-specific |
| Centrifuge | 1 | 25% | 111 | LAMP-specific |
| PCR machine***** | 1 | 15% | 82 | LAMP-specific |

* Used for LAMP QA

**some capital equipment was shared
